# Increased Respiratory Disease Mortality at a Microwave Popcorn Production Facility with Worker Risk of Bronchiolitis Obliterans

**DOI:** 10.1371/journal.pone.0057935

**Published:** 2013-02-28

**Authors:** Cara N. Halldin, Eva Suarthana, Kathleen B. Fedan, Yi-Chun Lo, George Turabelidze, Kathleen Kreiss

**Affiliations:** 1 Epidemic Intelligence Service Program, Centers for Disease Control and Prevention, Atlanta, Georgia, United States of America; 2 Division of Respiratory Disease Studies, National Institute for Occupational Safety and Health, Morgantown, West Virginia, United States of America; 3 Missouri Department of Health and Senior Services, Jefferson City, Missouri, United States of America; Universität Bochum, Germany

## Abstract

**Background:**

Bronchiolitis obliterans, an irreversible lung disease, was first associated with inhalation of butter flavorings (diacetyl) in workers at a microwave popcorn company. Excess rates of lung-function abnormalities were related to cumulative diacetyl exposure. Because information on potential excess mortality would support development of permissible exposure limits for diacetyl, we investigated respiratory-associated mortality during 2000–2011 among current and former workers at this company who had exposure to flavorings and participated in cross-sectional surveys conducted between 2000–2003.

**Methods:**

We ascertained workers' vital status through a Social Security Administration search. Causes of death were abstracted from death certificates. Because bronchiolitis obliterans is not coded in the International Classification of Disease 10^th^ revision (ICD-10), we identified respiratory mortality decedents with ICD-10 codes J40–J44 which encompass bronchitis (J40), simple and mucopurulent chronic bronchitis (J41), unspecified chronic bronchitis (J42), emphysema (J43), and other chronic obstructive pulmonary disease (COPD) (J44). We calculated expected number of deaths and standardized mortality ratios (SMRs) with 95% confidence intervals (CI) to determine if workers exposed to diacetyl experienced greater respiratory mortality than expected.

**Results:**

We identified 15 deaths among 511 workers. Based on U.S. population estimates, 17.39 deaths were expected among these workers (SMR = 0.86; CI:0.48-1.42). Causes of death were available for 14 decedents. Four deaths among production and flavor mixing workers were documented to have a multiple cause of ‘other COPD’ (J44), while 0.98 ‘other COPD’-associated deaths were expected (SMR = 4.10; CI:1.12–10.49). Three of the 4 ‘other COPD’-associated deaths occurred among former workers and workers employed before the company implemented interventions reducing diacetyl exposure in 2001.

**Conclusion:**

Workers at the microwave popcorn company experienced normal rates of all-cause mortality but higher rates of COPD-associated mortality, especially workers employed before the company reduced diacetyl exposure. The demonstrated excess in COPD-associated mortality suggests continued efforts to lower flavoring exposure are prudent.

## Introduction

Fixed obstructive lung disease caused by inhalation of butter flavoring chemicals, specifically diacetyl, was first recognized in 2000 among former workers of a microwave popcorn manufacturing plant located in Missouri. At this plant, eight former workers were diagnosed with clinical or pathological bronchiolitis obliterans, an irreversible lung disease[1], [2], [3], [4]. Findings from surveys conducted by the National Institute for Occupational Safety and Health (NIOSH) during 2000–2003 indicated that current workers in this facility had a 27% prevalence of spirometric abnormalities and 2.3-fold the expected rate of spirometric abnormalities compared to the general U.S. population, adjusting for age, race, gender, and smoking status[5].

Following the description of these eight sentinel cases, bronchiolitis obliterans has been recognized in workers throughout the microwave popcorn industry, flavoring manufacturing industry, and among workers involved in the chemical synthesis of diacetyl[6], [7], [8], [9], 10. Studies involving laboratory animals have demonstrated that exposure to butter flavoring chemicals, and specifically diacetyl and 2, 3-pentanedione, causes damage to the respiratory tract that is consistent with the development of bronchiolitis obliterans[11], [12], [13].

NIOSH has drafted a recommended exposure limit of 5 parts per billion (ppb) for diacetyl (2,3-butanedione) and 9.3 ppb for 2,3-pentanedione, a structurally similar substitute, both as a time-weighted average (TWA) during a 40-hour work week[14]. The risk assessment supporting the diacetyl recommended exposure limit used a job-exposure matrix and morbidity data for the Missouri plant's current and former workers to calculate both prevalence and incidence of pulmonary function impairment in relation to cumulative exposure to diacetyl over a 45-year working lifetime. Mortality implications of pulmonary function abnormalities or decline used in estimating incidence of pulmonary function impairment are largely derived from populations affected by cigarette smoking[15], [16], [17], and it is uncertain whether smoking-related mortality is similar to the natural history of bronchiolitis obliterans associated with flavorings exposure. Thus, the robustness of the diacetyl risk assessment would be enhanced if excess mortality were demonstrated for a diacetyl-exposed worker population.

Most mortality studies of working populations are conducted to investigate long-latency diseases such as suspected occupational malignancies. As such, they study rare disease mortality in cohorts that are large and in which cohort members have decades of follow up to fulfill latency requirements. In contrast, flavoring-exposed cohorts are at risk for short-latency respiratory impairment, which is irreversible, but may stabilize with cessation of inhalation exposure to flavorings[4]. The abnormal pulmonary function tests that could result in increased mortality were not rare among those current and former workers surveyed at the Missouri microwave popcorn manufacturing plant studied by NIOSH. The public health urgency of establishing recommended and permissible exposure limits prompted our looking at early mortality within the cohort of current and former workers of the Missouri plant. The basis for this cohort of 534 workers was a series of cross-sectional studies among current and former workers in the early 2000 s, conducted to screen for pulmonary disease.

## Methods

We conducted this study with the review and approval of the National Institute for Occupational Safety and Health (NIOSH) Human Subjects Review Board (protocol number HSRB-11-DRDS-01XP) which granted a waiver of informed consent for information obtained from questionnaires.

### Study population

From November 2000 through August 2003, we performed eight cross-sectional surveys at the Missouri popcorn manufacturing plant at four-six month intervals, enrolling 373 current workers (average participation rate of 80%)[18]. Additionally, through efforts of the Missouri Department of Health and Senior Services (MoDHSS), we enrolled 161 former workers through newspaper advertisements, referral by current workers, and telephone invitations to contract workers whose names were supplied by two agencies that provided the plant with temporary employees. A majority of former workers were enrolled in November 2000, however former workers continued to be enrolled through August 2003. We estimated that the company had employed approximately 425 temporary and permanent workers between 1992 and 2000 who had left employment[3], for a former worker participation rate of 38%. All current and former workers who participated in the 2000–2003 NIOSH surveys were eligible for this cohort mortality follow up. The overall participation rate of the current workers in the cross-sectional surveys, and therefore in this mortality follow up, could not be determined because employment rosters were not available from the manufacturing company at the time of the surveys. However, counts of current and contract employees were provided at the time of each survey, and participation ranged from 71%–91% across the eight NIOSH surveys[19].

During the cross-sectional surveys, NIOSH investigators obtained written informed consent from all participants prior to interview and spirometry testing. Trained interviewers administered a standardized questionnaire, including work history, described previously[1], as well as previous jobs and previous exposures. We compared spirometry test results, collected according to professional thoracic society guidance[20], to lower limits of normal derived from the Third National Health and Nutrition Examination Survey[21]. We defined spirometric obstruction as FEV_1_ and FEV_1_/FVC both below their lower limits of normal, spirometric restriction as FVC below the lower limit of normal, and a mixed pattern of obstruction and restriction when all three values fell below their lower limits of normal.

We were most interested in analyzing the subset of workers who worked in positions with potential for exposures to flavorings (511/534 workers, 95.7% of the cohort). Those with potential flavoring exposure worked in the (1) microwave popcorn production area which includes the mixing room and/or packaging lines; (2) the quality control laboratory, where workers continually popped bags of microwave popcorn (approximately 100 bags per worker per shift)[19]; and/or were (3) maintenance and warehouse workers who worked around the indoor production processes. Those with high risk from flavorings exposure were workers who worked in the mixing room, quality control laboratory, and/or in maintenance. Among those who held jobs with potential flavorings exposure, we defined subcohort 1 as the 155 former workers and the 136 workers currently employed in November 2000 who may have had similar ranges of exposures prior to company efforts to lower exposures. The 220 workers in subcohort 2 were hired after November 2000 and joined the cohort in any of the seven subsequent surveys of current workers conducted from April 2001 through August 2003 during which time the company made interventions to lower exposure[18].

Cumulative and average diacetyl exposure estimates for workers were made by calculating average diacetyl exposure by job in the calendar period worked, weighted by time worked in that job and calendar period. Exposures prior to November 2000 were assumed to be those measured by NIOSH during the exposure survey in November 2000. All measurements were adjusted for relative humidity and days from sample collection to extraction, subsequently shown to be necessary for the NIOSH 2557 method for measuring diacetyl concentrations from air samples used during the 2000–2003 industrial hygiene surveys[14], [22]. Average exposures were calculated by dividing cumulative exposures by tenure.

### Vital status follow-up and cause of death coding

Vital status of the worker cohorts was ascertained through two preliminary searches of Social Security Administration vital status records conducted for deaths occurring January 1, 2001 to November 30, 2011 and was not linked with National Death Index information. The first search matched on first and last name, and birth year and month. The second search matched on last name and complete date of birth (month, day, and year). The results generated by these two searches were then verified using other attributes collected from the workers during the 2000–2003 surveys. MoDHSS abstracted the underlying and contributing causes of death listed on the death certificates. All causes of death were coded by a National Center for Health Statistics nosologist according to the Tenth Revision of the International Classification of Diseases (ICD-10)[23].

Bronchiolitis obliterans is not a recognized disease classification in the ICD-10. Because it is a disease often characterized by fixed airways obstruction, we assumed the most probable code for decedents who may have had this disease to be ICD-10 code J44, ‘other chronic obstructive pulmonary disease (COPD)’.

### Statistical analysis

The Wilcoxon Rank Sum Test was used to test differences in median values between two groups and the Kruskal-Wallis Test was used to test differences in median values between three groups. All categorical data was analyzed by using Chi-square and Fisher's exact tests. All statistical analyses were generated using SAS/STAT® software, Version 9.2 of the SAS System for Windows (SAS Institute Inc., Cary, NC, USA). We used a probability (*p*) of 0.05 for statistical significance.

We compared all-cause and COPD mortality with expected mortality in the U.S. population, adjusting for age, gender, race, and calendar interval, using the NIOSH Life Table Analysis Software (LTAS) with underlying and multiple cause of death referent rates^24^,^25^. Person-years were tabulated by LTAS from the date of initial employment at the microwave popcorn plant (obtained from questionnaire interview) until the time each worker died or until the end of the follow up period (November 30, 2011).

LTAS software utilizes U.S. mortality rates for 119 causes of death categories [26], [27]. These categories, created by NIOSH, may correspond to one or multiple ICD-10 codes. Our analysis focused on cause of death category ‘Minor 66 Chronic Obstructive Pulmonary Disease’. Minor 66 includes ICD-10 codes J40–J44, which encompass bronchitis (J40), simple and mucopurulent chronic bronchitis (J41), unspecified chronic bronchitis (J42), emphysema (J43), and other chronic obstructive pulmonary disease (COPD) (J44). Because bronchiolitis obliterans clinically fits most closely into ICD-10 code J44 (other COPD), we created a custom rate file to target multiple cause mortality rates for J44 specifically.

Standardized mortality ratios (SMRs) were calculated to identify if this cohort of microwave popcorn workers exposed to flavorings experienced excess all-cause and COPD (cause of death category ‘Minor 66’, and J44 specifically) mortality than expected, based on the U.S. population experience. We used multiple-cause analysis, which includes the underlying cause of death as well as any contributory causes listed on the death certificate. This type of analysis is ideal for diseases, which may not be the underlying cause of death, but are significant enough to be listed on the death certificate. A single decedent with a death certificate listing two causes of death included in a death category (such as ‘Minor 66’) in the U.S. rate files contributes two counts of death in LTAS multiple cause analysis[25].

## Results

### Study population

The former workers and current workers in November 2000 (before company interventions) were very similar in age, race/ethnicity, smoking status and prevalence of abnormal spirometric results on their initial test (Table 1). Despite having similar rates of spirometric abnormalities of obstruction, restriction, and mixed obstructive-restrictive pattern, the former workers reported chest symptom prevalences that were 14–24% higher compared to workers currently employed in November 2000. Cumulative diacetyl exposure and tenure were significantly lower among former workers (median diacetyl exposure = 2.35 parts per million [ppm] years; median tenure = 12 months) than workers currently employed in November 2000 (median diacetyl exposure = 6.95 ppm years; median tenure = 58.2 months). The former worker group included eight sentinel cases of bronchiolitis obliterans, half of whom were on lung transplant lists[4], but comparably low percent predicted FEV_1_ existed among current workers in November 2000 as among those on lung transplant lists.

**Table 1:** Characteristics of former and two current worker groups constituting Subcohort 1 before interventions and Subcohort 2 after interventions began to lower exposure.

		Subcohort 1			Subcohort 2	
		Before Interventions			After Interventions	
		Former Workers	Current Workers		Current Workers	
			in 2000		in 2001–03	
Characteristics		n = 155	n = 136	p-value^a^	n = 220	p-value^b^
Median age, years		32.0	34.0	0.52	25.0	<0.0001
	Min-Max	20–62	18–67		18–63	
Male gender, n (%)		54 (34.8)	64 (47.1)	0.04	128 (58.2)	<0.0001
White race/ethnicity, n (%)		146 (94.2)	122 (89.7)	0.22	154 (70.0)	<0.0001
Hispanic race/ethnicity, n (%)		9 (5.8)	13 (9.6)		65 (29.5)	
Ever smoking status, n (%)		99 (63.9)	79 (58.1)	0.34	140 (63.6)	0.58
Never smoking status, n (%)		56 (36.1)	57 (41.9)		80 (36.4)	
Ever worked in a high risk job, n (%)		11 (7.1)	33 (24.3)	<0.0001	11 (5.0)	0.0003
Median tenure, months		12.0	58.2	<0.0001	4	<0.0001
	Min–Max	0.03–189.6	1.0–243.9		0.03–31.2	
Median cumulative diacetyl exposure, ppm-yrs		2.35	6.95	<0.0001	0.05	<0.0001
	Min–Max	0.01–65.26	0.09–52.64		0–1.02	
Median average diacetyl exposure, ppm		2.68	1.89	<0.0001	0.25	<0.0001
	Min–Max	0.12–9.74	0.08–9.89		0.002–1.89	
Median follow up, months		133.0	133.1	<0.0001	115.9	<0.0001
	Min–Max	6.1–145.0	51.2–133.2		14.8–128.1	
Usual cough^c^, n (%)		77 (49.7)	49 (36.0)	0.02	41 (18.6)	<0.0001
Shortness of breath^d^, n (%)		80/152 (52.6)	37/131 (28.2)	<0.0001	21/219 (9.6)	<0.0001
PFT results^e^, n (%)						
	Obstruction	10 (6.5)	11 (8.1)	0.42	5 (2.3)	<0.0001
	Restriction	23(14.8)	12 (8.8)		15 (6.8)	
	Mixed obstructive-restrictive pattern	17 (11.0)	11 (8.1)		3 (1.4)	
	Non interpretable test or no test	2 (1.3)	3 (2.2)		4 (1.8)	

a p-values for tests of equality of medians or prevalence comparing Former Workers and Current Workers employed in 2000.

b p-values for tests of equality of medians or prevalence comparing Subcohort 1 and Subcohort 2.

c Positive response to "Do you usually have a cough?"

d Positive response to "Do you get short of breath walking with people of your own age on level ground?"

e Pulmonary function test (PFT) interpretation of each worker's initial test.

In contrast, the current workers hired after the company began to lower exposures, subcohort 2, differed from subcohort 1 in virtually every demographic, exposure, respiratory symptom, and spirometric category. Subcohort 2 was younger (median age was eight years younger than subcohort 1), was predominantly male, and included more Hispanics. They were much less symptomatic, and rates of spirometric abnormalities were low. Subcohort 2 had shorter tenure both because questionnaire follow-up was limited to 2.5 years maximum from hire, and turnover was high. For most of subcohort 2, their diacetyl exposures fell below detection limits during the 2.5 years of follow-up[18] with median cumulative diacetyl exposures of 0.05 ppm years.

Prevalences of jobs and exposures prior to employment at the Missouri plant reported by survey participant groups are presented in Table 2. Subcohort 1 had a significantly higher proportion of those who responded that they had previously been exposed to grain dust, when compared to subcohort 2. Additionally, significantly more former workers reported that they had been exposed to a chemical or substance that affected their breathing, when compared to current workers in 2000. Significantly more workers in subcohort 1, compared to subcohort 2, reported exposure to a chemical or substance that affected their breathing; however this relationship was most likely driven by the increased proportion of former workers in subcohort 1 who answered ‘yes’ to this question.

**Table 2:** Reported jobs and exposures prior to employment at Missouri microwave popcorn manufacturing plant.

	Subcohort 1			Subcohort 2	
	Before Interventions			After Interventions	
	Former Workers	Current Workers		Current Workers	
		in 2000		in 2001-03	
Jobs and Exposuresa,b	n = 155	n = 136	p-value^c^	n = 220	p-value^d^
Mining^a^, n (%)	3 (1.9)	2 (1.5)	1.00	6 (2.7)	0.54
Farming^a^, n (%)	49 (31.6)	48 (35.3)	0.51	70 (31.8)	0.71
Chemical manufacturing like explosives, dyes, lacquers, and celluloid^a^, n (%)	16 (10.3)	9 (6.6)	0.26	26 (11.8)	0.23
Exposed to fire smoke^b^, n (%)	17 (11.0)	12 (8.8)	0.54	23 (10.5)	0.86
Exposed to irritant gases like chlorine, sulfur dioxide, ammonia, and phosgene^b^, n (%)	24 (15.5)	24 (17.6)	0.62	47 (21.4)	0.16
Exposed to mineral dusts including coal, silica, and talc^b^, n (%)	10 (6.5)	3 (2.2)	0.08	12 (5.5)	0.61
Exposed to grain dusts^b^, n (%)	48 (31.0)	40 (29.4)	0.77	44 (20.0)	0.01
Exposed to oxides of nitrogen including silo gas^b^, n (%)	5 (3.2)	10 (7.4)	0.11	5 (2.3)	0.10
Exposed to asbestos^b^, n (%)	9 (5.8)	11 (8.1)	0.44	17 (7.7)	0.71
Exposed to any chemical or substance that affected your breathing^b^, n (%)	33 (21.3)	17 (12.5)	0.05	23 (10.5)	0.03

a Positive response to "Have you ever worked in…?"

b Positive response to "Have you been…?"

c p-values for tests of equality of prevalence comparing Former Workers and Current Workers employed in 2000.

d p-values for tests of equality of prevalence comparing Subcohort 1 and Subcohort 2.

### Decedents

We found 15 decedents among the entire cohort, 12 of whom belonged to the 291 members of subcohort 1 (Table 3). All decedents worked in flavorings-exposed areas of the production plant. Our collaborators from MoDHSS could not locate the death certificate for one decedent ascertained through Social Security Administration records; we assigned the ICD-10 code for ‘unknown cause of mortality’ (code R99) to this decedent. Death certificates were available for the remaining 14 decedents who died in Missouri. MoDHSS contacted the treating physicians of two decedents to confirm the contributing cause of deaths listed on the certificate. Autopsy was not performed on any decedent.

**Table 3:** Characteristics of COPD decedents and the remaining cohort members.

		COPD Decedents	Non-COPD Decedents	Non-Decedents	
Characteristics		n = 4	n = 11	n = 496	p-value^a^
Belong to Subcohort 1, n (%)		3 (75.0)	9 (81.8)	279 (56.2)	0.19
Belong to Former worker group, n (%)		2 (50.0)	6 (54.5)	147 (29.6)	0.26
Median age, years		49.5	45.0	30.0	0.0015
Median age at death, years		56.5	48.0		0.08
Ever worked in a high risk job, n (%)		1 (25.0)	0	54 (10.9)	0.32
Median tenure, months		17.2	23.9	9.1	0.54
	Min–Max	0.3–30.0	0.5–121.8	0.03–243.9	
Median cumulative diacetyl exposure, ppm-yrs		3.29	5.34	0.57	0.07
	Min–Max	0–24.33	0.02–25.11	0–65.26	
Median average diacetyl exposure, ppm		1.99	2.20	0.37	0.09
	Min–Max	0.004–9.74	0.37–2.70	0.002–9.89	
Ever smoking status, n (%)		2 (50.0)	9 (81.8)	307 (61.9)	0.42
Never smoking status, n (%)		2 (50.0)	2 (18.2)	189 (38.1)	
Usual cough^b^, n (%)		3 (75.0)	5 (45.5)	159 (32.1)	0.10
Shortness of breath^c^, n (%)		2 (50.0)	3 (27.3)	133 (27.3)	0.63
PFT results^d^ (n = 511), n (%)					
	Obstruction	1 (25.0)	1 (9.1)	24 (4.8)	0.0009
	Restriction	0	1 (9.1)	49 (9.9)	
	Mixed obstructive-restrictive pattern	2 (50.0)	2 (18.2)	27 (5.4)	
	Non interpretable test or no test	1 (25.0)	0	8 (1.6)	

a p-values for tests of equality of medians or prevalence comparing COPD decedents, Non-COPD decedents and Non-decedents.

b Positive response to "Do you usually have a cough?"

c Positive response to "Do you get short of breath walking with people of your own age on level ground?"

d Pulmonary function test (PFT) interpretation of each worker's initial test.

Of the 14 decedents with known causes of death, 5 were found to have a respiratory cause of death. One decedent had two contributing respiratory causes of death including respiratory failure (J96.9) and pneumonia (J18.9). Four other decedents had contributing or underlying cause of death from COPD (LTAS cause of death category ‘Minor 66’). Specifically, two of these four decedents were determined to have chronic obstructive pulmonary disease, unspecified (J44.9), as a multiple cause of death. The third decedent's death certificate listed end-stage bronchiolitis obliterans as the underlying cause of death and was assigned ICD-10 code J44.8 (other specified chronic obstructive pulmonary disease). The death certificate of the fourth decedent listed two contributing respiratory causes of death: lung damage (coded as J43.9 ‘unspecified emphysema’) and COPD related to exposures to flavoring in the popcorn plant (coded as J44.9 ‘unspecified COPD’).

The expected number of all-cause deaths among the entire cohort based upon U.S. referent rates for underlying cause of death was 17.39 (SMR = 0.86; 95% confidence interval [CI]: 0.48–1.42). The expected number of COPD-associated deaths (ICD-10 codes J40–J44) among the entire cohort based upon U.S. referent rates for multiple-cause mortality was 1.16, where five COPD-associated multiple causes of death (among four decedents) were observed among the entire cohort (SMR = 4.30; CI: 1.40–10.04). The expected number of ‘other chronic obstructive pulmonary disease’ associated deaths (ICD-10 code J44) among the cohort based upon U.S. referent rates for multiple-cause mortality was 0.98, where four deaths were observed (SMR = 4.10; CI: 1.12–10.49) (Table 4).

**Table 4:** Standardized mortality ratios (SMR) for microwave popcorn production workers.

Group	Disease and ICD-10^1^ Code	Underlying or Multiple Cause Mortality Analyses	Obs.^2^	Exp.^3^	SMR	95% CI
Entire Cohort	All-Cause	Underlying	15	17.39	0.86	0.48–1.42
Entire Cohort	All-Cause	Multiple	34	42.08	0.81	0.56–1.13
Entire Cohort	COPD (J40–J44)	Multiple	5	1.16	4.30	1.40–10.04
Subcohort 1	COPD (J40–J44)	Multiple	4	0.95	4.22	1.15–11.79
Entire Cohort	Other COPD (J44)	Multiple	4	0.98	4.10	1.12–10.49
Entire Cohort	Ischemic Heart Disease (I20–I22, I24–I25, I51.3, I51.6)	Underlying	4	2.28	1.75	0.48–4.88
Entire Cohort	Ischemic Heart Disease (I20–I22, I24–I25, I51.3, I51.7)	Multiple	4	4.12	0.97	0.26–2.47

1 World Health Organization International Classification of Diseases Code 10th revision.

2 Observed number of causes of death.

3 Expected number of causes of death.

Subcohort 1, which experienced greater cumulative and average diacetyl exposure, had three deaths with a total of four COPD-associated multiple causes of death. The expected number of COPD-associated deaths among subcohort 1 was 0.95 (SMR = 4.22; CI: 1.15–11.79). In contrast, COPD-associated SMR was not significantly elevated for subcohort 2.

The most common cause of death among the entire cohort was acute myocardial infarction (I21.9). This cause falls under cause of death category ‘Minor 55 ischemic heart disease’. SMRs for underlying and multiple-cause mortality analysis for Minor 55 were not significantly elevated among the entire cohort (underlying SMR = 1.75, CI: 0.48–4.88; multiple-cause SMR = 0.97, CI: 0.26–2.47).

All COPD decedents had worked on the microwave popcorn production line as packers or as flavor mixers. Three of the four COPD decedents participated in pulmonary function testing (spirometry) during at least one of the NIOSH surveys. All three were found to have abnormal lung function on their initial NIOSH exam during the 2000–2003 period, with one case of obstruction and two cases with mixed obstructive-restrictive pattern. The 11 remaining decedents all participated in spirometry and four (36.4%) were found to have abnormal lung function compared to 20.4% of the remainder of the cohort (n = 491) who participated in spirometry testing (*p* = 0.25) (Table 3). There were no differences among COPD-decedents, non-COPD decedents, and non-decedents with respect to previous jobs or previous exposures (data not shown).

## Discussion

Previous descriptions of these microwave popcorn company workers documented cases of lung disease consistent with bronchiolitis obliterans, excessive spirometric obstruction, accelerated decline in FEV_1_ during employment, and diacetyl exposure-related health outcomes[1], [3], [4], [18]. This mortality study now suggests that these workers experience an increase in COPD-associated deaths, compared to the U.S. population. Overall mortality was not statistically different from that predicted for the age, gender, and ethnic distribution of the worker cohort, which remains young with a median age of about 52 after about 11 years of mortality follow up.

Workers with an abnormal spirometric result ranged across the cohort from 12.3% in subcohort 2 to 33.5% in the former worker group of subcohort 1. This trend mirrors the average diacetyl exposure experienced in each group, with subcohort 2 workers experiencing the lowest average exposure, followed by current workers from 2000, and former workers.

Current workers from 2000 and 2001–2003 reported respiratory symptoms at lower rates compared to former workers. A majority of former workers reported being troubled by shortness of breath on exertion and usual cough. The rates for respiratory symptoms experienced by former workers and current workers tested in 2000 were higher than rates of abnormal spirometry results for these same groups. This trend has also been documented in biopsy-confirmed case series among mustard gas-exposed patients from the Iran-Iraq war, some U.S. soldiers returning from Iraq and Afghanistan, and other case series where many patients have normal spirometric results but report respiratory symptoms[28], [29], [30].

An excess of COPD-associated mortality was found among the entire cohort and specifically among subcohort 1, which experienced higher exposure to diacetyl before the company introduced interventions. COPD decedents had somewhat shorter median tenure, lower cumulative diacetyl exposure and lower average diacetyl exposure compared to non-COPD decedents, but the combined decedent values were much higher than non-decedents. Diacetyl-associated lung function impairment has a short latency, and affected workers may drop out of the workforce before accumulating substantial cumulative exposure. The risk for bronchiolitis obliterans may be associated with short-term peak exposures to butter flavorings[6], and average exposures may be more pertinent to risk than cumulative exposures. Perhaps, for these reasons, we did not find exposure-response relationships for COPD mortality among the decedents. However, criteria for a causal association of diacetyl with respiratory disease have been met in the body of scientific work in exposed workers and animal experiments[14].

Finally, the COPD decedents who were tested had abnormal spirometry results, which have previously been associated with diacetyl exposure[1]. Additionally, even though rates of ever smoking in this cohort were higher (*p*<0.0001) than the U.S. average during a similar time period (U.S. ever smoking prevalence was 45.6% in 1999–2001 and 43.3% in 2002–2004)[31], two COPD decedents had never smoked, eliminating the most common cause of COPD mortality. These findings suggest that limiting diacetyl exposure might reduce COPD-associated mortality, supporting efforts to establish a recommended exposure limit.

### Limitations

Because only 38% of former workers participated in spirometry, we are unclear how representative our findings are of all workers who were employed prior to November 2000. Increased symptom rates, well out of proportion to their prevalences of ever-smoking, which were not statistically different, may have motivated the former workers' participation in NIOSH medical testing and may not have reflected the health of all former workers, the majority of whom could not be located or identified. We do not have information about participants' jobs or exposures following the 2000–2003 surveys. We are missing the underlying and contributing causes of death for one decedent outside of Missouri, and did not pursue locating the state holding the death certificate through the National Death Index. This decedent was a current worker in subcohort 1 and had reversible mild airway obstruction in 2000. Long-term follow up of this cohort using the National Death Index, which provides more complete but less timely death registration, will be necessary to improve estimates of excess COPD deaths.

Bronchiolitis obliterans is not recognized in the ICD-10 classification system, potentially resulting in misclassification of causes of death. Additionally, previous epidemiologic studies of this cohort describe workers with airways obstruction receiving physician diagnoses of chronic bronchitis and asthma prior to 2000[1]. U.S. mortality rates for NIOSH cause of death category Minor 66 ‘Chronic Obstructive Pulmonary Disease' (ICD-10 codes J40–J44) and/or ICD-10 code J44 specifically, may underestimate causes of deaths potentially related to occupational flavoring exposure, as well as include obstructive causes of respiratory death unlikely to be related to workplace exposures. However, no decedent had asthma as a multiple or underlying cause of death, suggesting that prior misclassification of diagnoses did not result in an underestimate of deaths that may have reflected occupational disease.

End stage bronchiolitis obliterans was written as the underlying cause of death of one decedent who was also one of the eight sentinel cases that brought bronchiolitis obliterans to public health attention. The physician of the other sentinel case decedent listed lung damage and COPD related to exposures to flavoring in the microwave popcorn production plant as contributing causes of death. Therefore, it is unclear if COPD (ICD-10 codes J40–J44), specifically ‘other chronic obstructive pulmonary disease’ (J44), accurately captures bronchiolitis obliterans mortality, and autopsy studies were not available to evaluate misclassification of causes of death.

Despite these limitations, our finding of excess standardized respiratory mortality, coded as COPD, documents that flavoring-related lung disease has potentially changed the causes of death in this comparatively young worker cohort, despite about one third of workers having left employment before enrollment or during follow-up. This is consistent with reports of many having been placed on lung transplant lists because of the severity of their physiologic abnormality [4] although none of the cohort is known to have undergone lung transplant. Long-term follow up of this cohort will more accurately reflect the ultimate burden of respiratory disease, cardiovascular disease associated with abnormal spirometry, and overall mortality.

The 11-year respiratory mortality excess in this microwave popcorn worker cohort underscores the importance of controlling inhaled butter flavoring exposures in food and flavoring production. Certainly, subcohort 2 was spared COPD mortality to date, but previous characterization of the newly-hired members of the cohort documented some excessive decline in FEV_1_ on serial testing[18] that, if continued, will result in spirometric abnormality. Despite short tenures at the time of the 2000–2003 surveys, average exposures remained about two orders of magnitude above NIOSH's recommended exposure limit for diacetyl, with a median time weighted average exposure of 1.89 ppm, nearly three orders of magnitude over the proposed exposure limit[13]. Follow-up characterization of exposures in subcohort 2 may be advantageous in interpreting future mortality findings of these less-exposed workers in the cohort.
